# Discontinuous microduplications at chromosome 10q24.31 identified in a Chinese family with split hand and foot malformation

**DOI:** 10.1186/1471-2350-14-45

**Published:** 2013-04-18

**Authors:** Li Dai, Ying Deng, Nana Li, Liang Xie, Meng Mao, Jun Zhu

**Affiliations:** 1 National Center for Birth Defects Monitoring, Chengdu, China; 2 Lab of Molecular Epidemiology of Birth Defects, Chengdu, China; 3 National office for Maternal and Child Health Surveillance, Chengdu, China; 4 Pulmonary Vascular Remodeling Research Unit, Chengdu, China; 5 Key Laboratory of Obstetric & Gynecologic and Pediatric Diseases and Birth Defects of Ministry of Education, West China Second University Hospital, Sichuan University, Chengdu, China

**Keywords:** SHFM, Microduplication, Han Chinese

## Abstract

**Background:**

Split hand/foot malformation (SHFM) is a congenital disorder characterized by a cleft of the hands and/or feet due to dificiency of central rays. Genomic rearrangement at 10q24 has been found to cause nonsyndromic SHFM (SHFM3).

**Methods:**

Four patients and fourteen unaffected individuals from a four-generation Chinese pedigree with typical SHFM3 phenotypes were recruited for this study. After informed consent was obtained, genome-wide copy number analysis was performed on all patients and two normal family members using the Affymetrix Cytogenetics Whole-Genome 2.7M Array. The results were then confirmed by real-time quantitative polymerase chain reaction in all available individuals of this pedigree. Candidate genes were further screened for mutation through sequence analyses.

**Results:**

Copy number analysis showed a microduplication at chromosome 10q24.31-q24.32 co-segregating with the SHFM phenotype. Compared to other known genomic duplications for SHFM3, the duplication described here contains two discontinuous DNA fragments. The minimal centromeric duplicated segment of 259 kb involves *LBX1*, *POLL* and a disrupted *BTRC*. The minimal telomeric duplication of 114 kb encompasses *DPCD* and one part of *FBXW4*. No coding and splice-site mutations of candidate genes in the region were found.

**Conclusions:**

Genomic duplications at chromosome 10q24.3, which were identified in the current study, provide further evidence for limb-specific cis-regulatory sequences in this region, highlighting the importance of chromosome 10q24.31-q24.32 in limb development and SHFM pathogenesis.

## Background

Split hand/foot malformation (SHFM) is a congenital disorder characterized by variable degrees of median clefts of hands and/or feet due to absence of the central ray digits. It also presents syndactyly, and aplasia/hypoplasia of the phalanges, metacarpals and metatarsals. SHFM shows highly variability in phenotypes between and/or within families, even between limbs of a single patient, ranging from mild syndactyly to severe central clefting of the autopods, oligodactyly or monodactyly [1]. The condition can occur as an isolated entity or a part of other anomalies, with an estimated incidence of 1/8,500 ~1/25,000 [2].

Currently, six loci for nonsyndromic SHFM phenotype have been identified: SHFM1 on 7q21 (OMIM 183600) [3], SHFM2 on Xq26 (OMIM 313350) [4-6], SHFM3 on 10q24 (OMIM 246560) [7-9], SHFM4 on 3q27 (OMIM 605289) [10], SHFM5 on 2q31 (OMIM 606708) [11,12] and SHFM6 on 12q13 (OMIM 225300) [13]. The autosomal dominant inherited SHFM1, 3 and 4 have been associated with deletions and translocations on 7q, genomic duplications on 10q24 and mutations in *TP63*, respectively. The only autosomal recessive SHFM6 have been found to be caused by mutations in *WNT10B*[13-15]. However, genetic causes for SHFM2 and SHFM5 are still unknown.

The SHFM3 locus was initially mapped to chromosome 10q24 by linkage analysis [7-9,16], then narrowed to a 0.5-Mb region through the identified genomic duplications involving *LBX1*, *BTRC* and *FBXW4*[17]. No intragenic mutations to date have been detected in candidate genes within or flanking duplicated regions. The relationship has been well established between SHFM3 and genomic duplications in 10q24 [17-20], a few were documented in Chinese patients [21] though a relative high birth prevalence rate of 0.64/10000 for nonsyndromic SHFM was reported [22]. In this study, we conducted an array-based genome-wide copy number variation (CNV) analysis on a Chinese family with SHFM and identified a discontinuous microduplication on 10q24.31-24.32 as the likely cause.

## Methods

### Subjects

**Figure 1:**
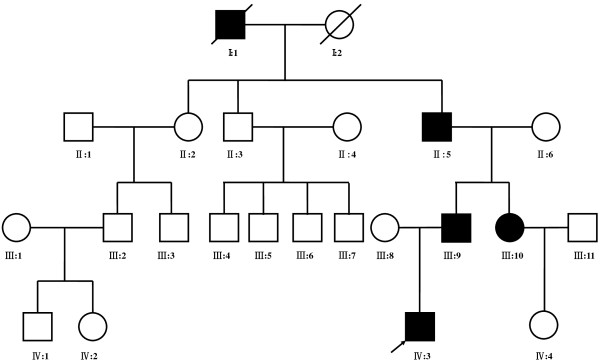
Pedigree chart of the Chinese SHFM family.

A SHFM family with four-generation-span was recruited after informed consent (Figure 1). Four affected (II:5, III:9, III:10 and IV:3) and 14 unaffected family members received full clinical evaluation and blood samples were collected for further studies. Clinical records and radiographic images were published under the patients’ written permission. This study was approved by the Research Ethics Committee of Sichuan University.

### Mutation screening

Genomic DNA was isolated from blood samples using a commercially available kit (Bio Teke, Beijing, China). As *TP63* is the only disease-causing gene for autosomal dominant SHFM, we first searched for possible mutations in the gene in family members as outlined previously [23]. We also screened candidate genes within the regions defined by array-based copy number variation analysis. Exons and their flanking intronic sequences of the *LBX1*, *BTRC*, *DPCD* and *FBXW4* were amplified by polymerase chain reaction (PCR). All the resulting products were purified, and sequenced bi-directionally using an ABI 3730xl sequencer. All primer sequences are available on request.

### Microarray analysis

Using the Affymetrix Cytogenetics Whole-Genome 2.7M array (Affymetrix Inc, USA), four affected and two unaffected individuals (II:6, III:8) in this family were selected to undertake CNV analysis. Experiments were carried out by Peking CapitalBio Company (Beijing, China) according to the manufacturer's protocol. The Affymetrix Chromosome Analysis Suite Software (ChAS) was used for genotype calling, quality control and CNV identification.

### Identification of the duplication by quantitative PCR

To confirm duplications at 10q24.31-q24.32 mapped by CNV analysis, six EvaGreen assays were designed to determine the relative copy number (RCN) for all family members. Primers for amplifying selected fragments in duplicated regions were described previously [20]. PCR conditions and locations of assays are presented in Additional file 1: Table S1. All reactions used 500nM of each primer, 10 ng of genomic DNA, and SsoFast EvaGreen Supermix quantitative PCR (qPCR) buffer (Bio-Rad, California, USA). PCRs were performed by using a Bio-Rad C1000™ Thermal Cycler (Bio-Rad, USA) in a 10-μl volume in 96-well plates with four replicates per sample. Reactions were run in a Bio-Rad CFX96™ Real-Time System with the following conditions: 98°C for 2 min, 98°C for 1 sec, annealing for 5 sec, and 40 cycles of 98°C 1 sec. The quantification of the target sequences was normalized to one assay from chromosome 11 [18], the RCN was determined on the basis of the comparative 2^-ΔΔCt^ method with a normal control DNA as the calibrator [24]. The experiments were repeated three times. A ~1.5-fold RCN was used for duplication. Relative DNA copy number was obtained by pair-wise comparisons of test and control DNAs.

## Results

### Clinical report

**Figure 2:**
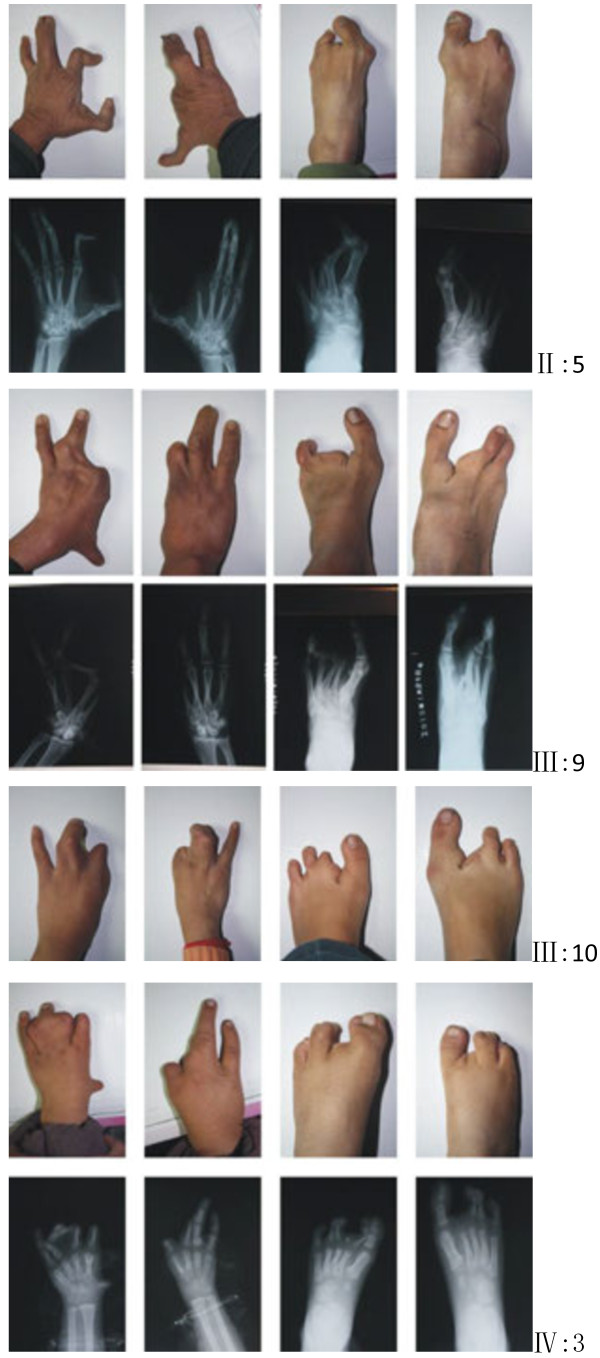
Clinical and X-ray photos of selected patients from this SHFM family.

Five individuals in this family were affected by SHFM with autosomal dominant transmission, but only four of them were available for this study (Figure 1). The proband (IV:3), one three-year-old boy, was affected by severe distal deficiency affecting all 4 limbs. Other three patients exhibited similar phenotypes including ectrodactyly of central digits, bilateral median cleft in feet, 3/4 finger or toe syndactly, hypoplasia of metacarpals and metatarsals. Representative clinical observations from photographs and X-ray data are summarized in Figure 2 and Additional file 2: Table S2. Notably, phenotypic variations were observed between affected family members. Triphalangeal thumb was only identified in patient II:5, and complete 1/2 toe syndactyly in individual IV:3. Similar hypoplasia/agenesis of 1^st^ ray existed in III:9, III:10 and IV:3. No non-limb malformations were identified in any patient.

### Mutation screening in candidate genes

Before genome-wide genotyping, we did not identify any mutation in exons of *TP63* gene using PCR and direct sequencing analysis. Also we sequenced four known genes in the critical regions at 10q24, and found no pathogenic mutation in the coding region of these genes.

### Copy number variation analysis

Using array-based genome-wide CNV, we identified similar duplications at 10q24.31-q24.32 in the four patients, but not in the two unaffected family members. The duplicated genomic contents contain two segments. The centromeric duplications, ranging from 247 kb to 260 kb, share a common overlapping segment containing *LBX1*, *POLL* and a disrupted *BTRC*. The telomeric duplications of 114–125 kb comprise *DPCD* and one part of *FBXW4* gene (Additional file 3: Table S3, Additional file 4: Figure S1). With use of qPCR assay, we confirmed the duplication co-segregating with SHFM phenotypes in the family. As predicted by ENCODE (http://www.genome.gov/10005107), this genomic region contains a variety of regulatory elements including enhancers, promoters and multiple transcription factor-binding sites. Particularly, the centromeric duplications involve two negative enhancers, hs324 and hs939, while the region between two duplications harbors a human limb-development-related positive enhancer, hs326 (chr10:103,266,649-103,267,972, http://enhancer.lbl.gov).

## Discussion

SHFM is a complicated limb malformation that shows highly phenotypic variability and genetic heterogeneity. Even though six SHFM loci have been mapped to date, causal genes for a large number of cases are still unknown. At the gene level, mutations in *TP63* and *WNT10B* can lead to autosomal dominant SHFM4 and autosomal recessive SHFM6, respectively. Recently a point mutation of *DLX5* gene was reported to be responsible for autosomal recessive SHFM [25]. Genomic rearrangements at 10q23-q24 have been identified in many familial or sporadic SHFM cases [17-20], the mechanisms remain to be elucidated. In current study, we identified a previously unreported copy number variation at 10q24.31-q24.32, segregating with SHFM in a four-generation Chinese family. The minimal duplicated genomic contents include a 247-kb fragment comprising *LBX1* and part of *BTRC*, and another fragment of 114 Kb containing *POLL*, *DPCD*, and disrupted *FBXW4*. Our findings expand the knowledge of genomic rearrangements leading to SHFM, and highlight the important role of chromosomal 10q24 region in regulating limb development.

A genotype-phenotype study showed that preaxial involvement of the upper limbs can be locus discriminators for SHFM3 [26]. In the current study, various preaxial upper limb anomalies were found in the affected individuals including proximal placed thumb, triphalangeal thumb, preaxial polydactyly, and absence of paraxial rays. In addition, more severe affected feet than hands, and worsening phenotype in patients of younger generations, were observed in the family. As noted in literature [9,19], the observed phenotypes strongly suggested that current disorder should be mapped to SHFM3 locus, chromosomal 10q24.

SHFM is considered a failure to maintain medial apical ectodermal ridge (AER) [1], so genes expressed in AER are reasonable candidates for SHFM3, such as the three known SHFM genes *TP63 ,WNT10B* and *DLX5.* Microduplications involving *LBX1*, *BTRC*, *POLL, DPCD* and one part of *FBXW4* (human *dactylin* gene) were shown for SHFM3 in two studies [17,19]. Another study reported that a 325-kb duplication containing *BTRC* and *POLL* leads to SHFM3, which is the smallest one identified to date [20]. A small number of genes at 10q24 like *BTRC*, *FBXW4*, *SUFU* and *FGF8* are also expressed in the developing limbs [1]. Among them, *FBXW4* has been considered as the best candidate gene due to its role in the SHFM3 ‘like’ *Dactylaplasia* mouse model [27,28]. *BTRC* is another promising candidate, because its product gets involved in essential pathways for limb development, such as the canonical WNT/β-catenin and NKFα signaling pathways. Despite the frequent observations of genomic rearrangements at 10q24 in SHFM patients, no exon mutations were found in these candidate genes.

In this study, the duplicated segments contain three entire genes (*LBX1, DPCD* and *POLL)* and one part of two genes (*BTRC* and *FBXW4*). The duplicated portion of *BTRC* gene contains its promoter and exon 1, while the duplicated segment of *FBXW4* includes 3^′^ end of the gene. Since *BTRC* and *FBXW4* are only partially duplicated in our SHFM cases, it is less likely that overexpression of these two gene causes SHFM. Comparing our findings with those of previous studies, *BTRC* is the most frequently identified gene involved in the genomic rearrangements. A duplication affecting *BTRC* and *POLL* only has been reported to be responsible for SHFM3 [20]. We speculate that limb-specific regulatory elements around or in *BTRC* gene may play an important role in SHFM pathogenesis, by controlling the expression of genes at 10q24 or the related downstream genes.

It is unclear how genomic rearrangement at this critical region causes the SHFM phenotype. The elevated expression of *BTRC* and *SUFU* in lymphoblastoid cells detected in SHFM3 patients suggested that the gene dosage (*BTRC*) or long-range control mechanisms (*SUFU*) may underlie overexpression [20]. In the *Dactylaplasia* mouse model, changes in the expression levels of *Lbx1, Btrc, Poll,* and *Dpcd* were not observed except for reduced levels of normal *Fbxw4* transcript [19], which implies duplications may disrupt normal expression of related genes. An increasing number of studies have shown that copy number variations are a common cause of human genetic disorders [29]. Duplications of regulatory elements have been described for human limb malformations. Brachydactyly type A2 is caused by a tandem duplication of a 5.5 kb region 3’ of *BMP2*[30]. Triphalangeal thumb-polysyndactyly syndrome is associated with duplications of ZPA regulatory sequence, a long range cis-regulator for *SSH* gene [31]. Based on current understanding of the molecular mechanisms for limb anomalies associated with duplications, the duplications described here may alter the dosage of a regulatory element involved in limb development or disrupt the co-expression domains, consequently leading to SHFM phenotypes.

## Conclusion

In summary, this report describes a discontinuous duplication at 10q24.3 responsible for a typical familial SHFM3. Our data suggest that chromosome 10q24.3 may contain key elements for regulating the coordinate expression of many genes for limb development. Future studies on the developmental effects of genomic rearrangements at this region in animal models would thus complement the human data presented here. Our findings could be of value to molecular diagnosis and deeper understanding of pathogenesis of this disorder.

## Abbreviations

SHFM: Split hand/foot malformation; BTRC: Beta-transducin repeat containing E3 ubiquitin protein ligase; LBX1: Ladybird homeobox 1; DPCD: Deleted in primary ciliary dyskinesia homolog (mouse); POLL: Polymerase (DNA directed), lambda; FBXW4: F-box and WD repeat domain containing 4; SUFU: Suppressor of fused homolog (Drosophila); BMP2: Bone morphogenetic protein 2.

## Competing interests

The authors declare that they have no competing interests.

## Authors’ contributions

LD studied the family, designed research plan and prepared manuscript. YD, NL and LX performed the molecular genetic studies. MM participated in clinical evaluation. JZ participated in collecting specimens. All authors reviewed the final manuscript and approved the publication of the clinical images. All authors read and approved the final manuscript.

## Pre-publication history

The pre-publication history for this paper can be accessed here:

http://www.biomedcentral.com/1471-2350/14/45/prepub

## Supplementary Material

Additional file 1: Table S1 Quantitatibe PCR Primers used in the study. Click here for file

Additional file 2: Table S2 Clinical features of four patients in the SHFM family. Click here for file

Additional file 3: Table S3 Genomic duplications at 10q21.31-q21.32 identified in SHFM patients. Click here for file

Additional file 4: Figure S1 Schematic overview of microduplications at chromosome 10q24.3 associated with SHFM. This graph was generated by the Affymetrix ChAS software. Click here for file

## References

[B1] Duijf PH van Bokhoven H Brunner HG Pathogenesis of split-hand/split-foot malformation Hum Mol Genet 2003 12 R51 R60 Spec No 1 10.1093/hmg/ddg090 12668597

[B2] Elliott AM Reed MH Chudley AE Chodirker BN Evans JA Clinical and epidemiological findings in patients with central ray deficiency: split hand foot malformation (SHFM) in Manitoba Canada Am J Med Genet A 2006 140 13 1428 1439 10.1002/ajmg.a.31245 16673359

[B3] Scherer SW Poorkaj P Allen T Kim J Geshuri D Nunes M Soder S Stephens K Pagon RA Patton MA Fine mapping of the autosomal dominant split hand/split foot locus on chromosome 7, band q21.3-q22.1 Am J Hum Genet 1994 55 1 12 20 8023840 PMC1918243

[B4] Faiyaz-Ul-Haque M Zaidi SH King LM Haque S Patel M Ahmad M Siddique T Ahmad W Tsui LC Cohn DH Fine mapping of the X-linked split-hand/split-foot malformation (SHFM2) locus to a 5.1-Mb region on Xq26.3 and analysis of candidate genes Clin Genet 2005 67 1 93 97 15617554 10.1111/j.1399-0004.2004.00369.x

[B5] Faiyaz U Haque M Uhlhaas S Knapp M Schuler H Friedl W Ahmad M Propping P Mapping of the gene for X-chromosomal split-hand/split-foot anomaly to Xq26-q26.1 Hum Genet 1993 91 17 19 8454282 10.1007/BF00230215

[B6] Ahmad M Abbas H Haque S Flatz G X-chromosomally inherited split-hand/split-foot anomaly in a Pakistani kindred Hum Genet 1987 75 2 169 173 10.1007/BF00591081 3817811

[B7] Gurrieri F Prinos P Tackels D Kilpatrick MW Allanson J Genuardi M Vuckov A Nanni L Sangiorgi E Garofalo G A split hand-split foot (SHFM3) gene is located at 10q24– > 25 Am J Med Genet 1996 62 4 427 436 10.1002/(SICI)1096-8628(19960424)62:4<427::AID-AJMG16>3.0.CO;2-Q 8723077

[B8] Nunes ME Schutt G Kapur RP Luthardt F Kukolich M Byers P Evans JP A second autosomal split hand/split foot locus maps to chromosome 10q24-q25 Hum Mol Genet 1995 4 11 2165 2170 10.1093/hmg/4.11.2165 8589697

[B9] Ozen RS Baysal BE Devlin B Farr JE Gorry M Ehrlich GD Richard CW Fine mapping of the split-hand/split-foot locus (SHFM3) at 10q24: evidence for anticipation and segregation distortion Am J Hum Genet 1999 64 6 1646 1654 10.1086/302403 10330351 PMC1377907

[B10] Celli J Duijf P Hamel BC Bamshad M Kramer B Smits AP Newbury-Ecob R Hennekam RC Van Buggenhout G van Haeringen A Heterozygous germline mutations in the p53 homolog p63 are the cause of EEC syndrome Cell 1999 99 2 143 153 10.1016/S0092-8674(00)81646-3 10535733

[B11] Boles RG Pober BR Gibson LH Willis CR McGrath J Roberts DJ Yang-Feng TL Deletion of chromosome 2q24-q31 causes characteristic digital anomalies: case report and review Am J Med Genet 1995 55 2 155 160 10.1002/ajmg.1320550204 7717414

[B12] Goodman FR Majewski F Collins AL Scambler PJ A 117-kb microdeletion removing HOXD9-HOXD13 and EVX2 causes synpolydactyly Am J Hum Genet 2002 70 2 547 555 10.1086/338921 11778160 PMC384929

[B13] Ugur SA Tolun A Homozygous WNT10b mutation and complex inheritance in Split-Hand/Foot Malformation Hum Mol Genet 2008 17 17 2644 2653 10.1093/hmg/ddn164 18515319

[B14] Khan S Basit S Zimri F Ali N Ali G Ansar M Ahmad W A novel homozygous missense mutation in WNT10B in familial split-hand/foot malformation Clin Genet 2012 82 1 48 55 10.1111/j.1399-0004.2011.01698.x 21554266

[B15] Blattner A Huber AR Rothlisberger B Homozygous nonsense mutation in WNT10B and sporadic split-hand/foot malformation (SHFM) with autosomal recessive inheritance Am J Med Genet A 2010 152A 8 2053 2056 10.1002/ajmg.a.33504 20635353

[B16] Raas-Rothschild A Manouvrier S Gonzales M Farriaux JP Lyonnet S Munnich A Refined mapping of a gene for split hand-split foot malformation (SHFM3) on chromosome 10q25 J Med Genet 1996 33 12 996 1001 10.1136/jmg.33.12.996 9004130 PMC1050809

[B17] de Mollerat XJ Gurrieri F Morgan CT Sangiorgi E Everman DB Gaspari P Amiel J Bamshad MJ Lyle R Blouin JL A genomic rearrangement resulting in a tandem duplication is associated with split hand-split foot malformation 3 (SHFM3) at 10q24 Hum Mol Genet 2003 12 16 1959 1971 10.1093/hmg/ddg212 12913067

[B18] Everman DB Morgan CT Lyle R Laughridge ME Bamshad MJ Clarkson KB Colby R Gurrieri F Innes AM Roberson J Frequency of genomic rearrangements involving the SHFM3 locus at chromosome 10q24 in syndromic and non-syndromic split-hand/foot malformation Am J Med Genet A 2006 140 13 1375 1383 16761290 10.1002/ajmg.a.31246

[B19] Kano H Kurosawa K Horii E Ikegawa S Yoshikawa H Kurahashi H Toda T Genomic rearrangement at 10q24 in non-syndromic split-hand/split-foot malformation Hum Genet 2005 118 3–4 477 483 16235095 10.1007/s00439-005-0074-0

[B20] Lyle R Radhakrishna U Blouin JL Gagos S Everman DB Gehrig C Delozier-Blanchet C Solanki JV Patel UC Nath SK Split-hand/split-foot malformation 3 (SHFM3) at 10q24, development of rapid diagnostic methods and gene expression from the region Am J Med Genet A 2006 140 13 1384 1395 16691619 10.1002/ajmg.a.31247

[B21] Yang W Hu ZJ Yu XF Li QH Zhang AJ Deng X Zhang AY Gao CS Liu Y Ao Y [A DNA duplication at chromosome 10q24.3 is associated with split-hand split-foot malformation in a Chinese family] Zhonghua Yi Xue Za Zhi 2006 86 10 652 658 16681918

[B22] Dai L Li YH Deng Y Zhu J Wang YP Liang J Zhang YW Liu ZY [Prevalence of congenital split hand/split foot malformation in Chinese population] Sichuan Da Xue Xue Bao Yi Xue Ban 2010 41 2 320 323 20506663

[B23] van Bokhoven H Hamel BC Bamshad M Sangiorgi E Gurrieri F Duijf PH Vanmolkot KR van Beusekom E van Beersum SE Celli J p63 Gene mutations in eec syndrome, limb-mammary syndrome, and isolated split hand-split foot malformation suggest a genotype-phenotype correlation Am J Hum Genet 2001 69 3 481 492 10.1086/323123 11462173 PMC1235479

[B24] Livak KJ Schmittgen TD Analysis of relative gene expression data using real-time quantitative PCR and the 2(−Delta Delta C(T)) Method Methods 2001 25 4 402 408 10.1006/meth.2001.1262 11846609

[B25] Shamseldin HE Faden MA Alashram W Alkuraya FS Identification of a novel DLX5 mutation in a family with autosomal recessive split hand and foot malformation J Med Genet 2012 49 1 16 20 10.1136/jmedgenet-2011-100556 22121204

[B26] Elliott AM Evans JA Genotype-phenotype correlations in mapped split hand foot malformation (SHFM) patients Am J Med Genet A 2006 140 13 1419 1427 16688749 10.1002/ajmg.a.31244

[B27] Sidow A Bulotsky MS Kerrebrock AW Birren BW Altshuler D Jaenisch R Johnson KR Lander ES A novel member of the F-box/WD40 gene family, encoding dactylin, is disrupted in the mouse dactylaplasia mutant Nat Genet 1999 23 1 104 107 10.1038/12709 10471509

[B28] Ianakiev P Kilpatrick MW Dealy C Kosher R Korenberg JR Chen XN Tsipouras P A novel human gene encoding an F-box/WD40 containing protein maps in the SHFM3 critical region on 10q24 Biochem Biophys Res Commun 1999 261 1 64 70 10.1006/bbrc.1999.0963 10405324

[B29] Zhang F Gu W Hurles ME Lupski JR Copy number variation in human health, disease, and evolution Annu Rev Genomics Hum Genet 2009 10 451 481 10.1146/annurev.genom.9.081307.164217 19715442 PMC4472309

[B30] Dathe K Kjaer KW Brehm A Meinecke P Nurnberg P Neto JC Brunoni D Tommerup N Ott CE Klopocki E Duplications involving a conserved regulatory element downstream of BMP2 are associated with brachydactyly type A2 Am J Hum Genet 2009 84 4 483 492 10.1016/j.ajhg.2009.03.001 19327734 PMC2667973

[B31] Lettice LA Heaney SJ Purdie LA Li L de Beer P Oostra BA Goode D Elgar G Hill RE de Graaff E A long-range Shh enhancer regulates expression in the developing limb and fin and is associated with preaxial polydactyly Hum Mol Genet 2003 12 14 1725 1735 10.1093/hmg/ddg180 12837695

