# Supplementary material for: Discontinuous microduplications at chromosome 10q24.31 identified in a Chinese family with split hand and foot malformation
Source: BMC Med Genet. 2013 Apr 18;14:45. doi: 10.1186/1471-2350-14-45 (PMC3637097; doi:10.1186/1471-2350-14-45)
Supplement: Additional file 1: Table S1 — Quantitatibe PCR Primers used in the study. [file 1471-2350-14-45-S1.docx]

**Supplementary table 1 Quantitative PCR Primers used in the study**

| **Primer** | **Locus** | **Primer sequence (5'-3')** | | **Amplicons position** | | **Annealing temperature (°C)** |
| --- | --- | --- | --- | --- | --- | --- |
|  |  | **Forward** | **Reverse** | **Start** | **Stop** |  |
| 38k upstream of *BTRC* | 10q24.31 | TGAACCCTCCCCCATCCT | GCTCAGTCCTTTTGATAAGTTAACCC | 103075359 | 103075431 | 56.9 |
| *BTRC* intron 1 | 10q24.32 | CAAAAATCCTCCCAGAACATCCT | TCATCATAGACAGGGATTAATTGTGG | 103135795 | 103135875 | 56.6 |
| *DPCD* | 10q24.32 | TGCGCAAGGACACCAAGAT | GAGACACTATAGACATCCTTAGGATAGGG | 103350960 | 103351035 | 61.5 |
| *FBXW4* exon 3 | 10q24.32 | CCTACCAGTTCCGTCCAGATG | GCCCAGCAAAGACTCCCAG | 103423293 | 103423355 | 54.8 |
| *FBXW4* exon 7 | 10q24.32 | CAGTGACTTTCCCCCAGGG | CGAACATAGGTGTCATAGCCACA | 103362111 | 103362200 | 56.9 |
| *KIRREL3* | 11q24.2 | GGGCCATCAGAGCTAAAGACC | CTTGGGGGAAGTGGAGGTTTA | 125800681 | 125800890 | 59.0 |

Amplicon position on the March 2006 human reference sequence NCBI Build 36.1.
