# Supplementary material for: Discontinuous microduplications at chromosome 10q24.31 identified in a Chinese family with split hand and foot malformation
Source: BMC Med Genet. 2013 Apr 18;14:45. doi: 10.1186/1471-2350-14-45 (PMC3637097; doi:10.1186/1471-2350-14-45)
Supplement: Additional file 2: Table S2 — Clinical features of four patients in the SHFM family. [file 1471-2350-14-45-S2.docx]

**Supplementary table 2. Clinical features of four patients in the SHFM family**

| **Patient No.** | **Gender** | **Left upper limb** | **Right upper limb** | **Left lower limb** | **Right lower limb** |
| --- | --- | --- | --- | --- | --- |
| Ⅱ:5 | Male | Proximal placed thumb; triphalangeal thumb; camptodactyly of 1^st^ and 2^nd^ finger; 3/4 syndactyly | Proximal placed thumb; camptodactyly of thumb; triphalangeal thumb; missing 2^nd^ finger; 3/4 cutaneous syndactyly | Camptodactyly of 1^st^ toe; absence of 2^nd^ toe; transversely placed proximal phalange of 3^rd^ toe; deficiency of middle, distal phalanges of 3^rd^ toe; cleft foot | Central cleft between 1/2 toe syndactyly and 3/4 toe syndactyly; absence of middle and distal phalanges of 3^rd^ toe; misplaced proximal phalanx of 2^nd^ toe |
| Ⅲ:9 | Male | Misplaced and hypoplasia 1^st^ metacarpus; missing thumb; absence of 2^rd^ finger; missing middle and distal phalanges of 3^rd^ finger; osteal fusion between 4^th^ finger and transversely placed proximal phalanges of 3^rd^ finger | Absence of 1^st^ ray and thumb; hypoplasia of 2^nd^ metacarpus; missing 2^nd^ finger; absence of middle, distal phalanges of 3^rd^ finger; nail aplasia of 4^th^ finger | Median cleft due to absence of 2^nd^ toe; missing distal phalanx of 3^rd^ toe; transversely placed proximal phalanx of 3^rd^ toe; 3/4 toe syndactyly; abnormal metatarsals; clinodactyly of 4^th^ toe | Absence of 2^nd^ and 3^rd^ toes; hypoplasia of 2^nd^ metatarsus; cleft foot |
| Ⅲ:10 | Female | Missing 1^st^ ray and thumb; absence of the middle, distal phalanges of 2^nd^ finger; 3/4 syndactyly | Absence of 1^st^ ray and thumb; deficiency of middle, distal phalanx of 2^nd^ finger; 3/4 syndactyly | Central cleft due to absence of 2^nd^ toe; hypoplasia of 2^nd^ metatarsus; missing distal phalanx of 3^rd^ toe; 3/4 toe syndactyly; cleft foot | Central cleft due to absence of 2^nd^ and 3^rd^ toes; hypoplasia of 2^nd^ metatarsus |
| Ⅳ:3 | Male | Duplicated distal phalanx of 4^th^ finger; missing 1^st^ metacarpus; proximal placed thumb remnant; missing middle and distal phalanges of 2^nd^, 3^rd^ fingers; 3/4 cutaneous syndactyly | Absence of 1^st^ metacarpus and thumb; missing middle, distal phalanges of 2^nd^ and 3^rd^ finger; cutaneous 3/4 syndactyly | 1/2 toe syndactyly; extra proximal phalanx of 2^nd^ toe; 3/4 toe syndactyly; median cleft | Cleft due to absence of 2^nd^ toe; 3/4 toe syndactly |
