# Supplementary material for: Discontinuous microduplications at chromosome 10q24.31 identified in a Chinese family with split hand and foot malformation
Source: BMC Med Genet. 2013 Apr 18;14:45. doi: 10.1186/1471-2350-14-45 (PMC3637097; doi:10.1186/1471-2350-14-45)
Supplement: Additional file 3: Table S3 — Genomic duplications at 10q21.31-q21.32 identified in SHFM patients. [file 1471-2350-14-45-S3.docx]

**Supplementary table 3. Genomic duplications at 10q21.31-q21.32 identified in SHFM patients**

| Patients | Segment 1 |  |  | Segment 2 |  |
| --- | --- | --- | --- | --- | --- |
|  | Genomic region* | Size (kbp) |  | Genomic region* | Size (kbp) |
| Ⅱ:5 | 102911736-103169477 | 257 |  | 103334900-103449414 | 114 |
| Ⅲ:9 | 102911736-103170849 | 259 |  | 103324414-103449414 | 125 |
| Ⅲ:10 | 102911736-103169477 | 257 |  | 103332499-103449414 | 116 |
| Ⅳ:3 | 102911736-103159334 | 247 |  | 103334900-103449414 | 114 |

*Based on the Affymetrix Cytogenetic 2.7M array data
