# Supplementary material for: Discontinuous microduplications at chromosome 10q24.31 identified in a Chinese family with split hand and foot malformation
Source: BMC Med Genet. 2013 Apr 18;14:45. doi: 10.1186/1471-2350-14-45 (PMC3637097; doi:10.1186/1471-2350-14-45)
Supplement: Additional file 4: Figure S1 — Schematic overview of microduplications at chromosome 10q24.3 associated with SHFM. This graph was generated by the Affymetrix ChAS software. [file 1471-2350-14-45-S4.docx]

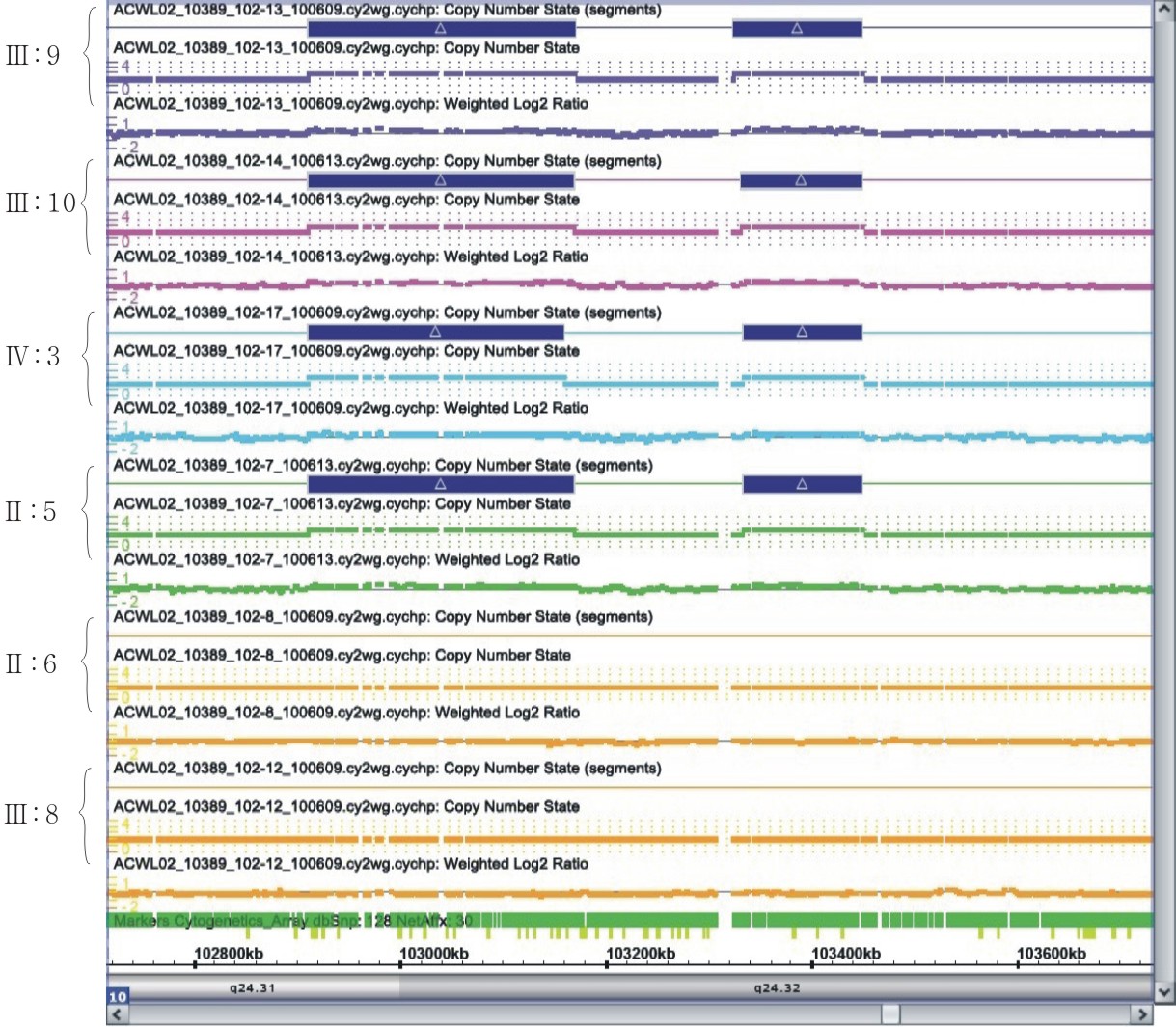


Supplementary figure 1. Schematic overview of microduplications at chromosome 10q24.3 associated with SHFM. This graph was generated by the Affymetrix ChAS software.
